# LncRNA DRAIC regulates cell proliferation and migration by affecting the miR-34a-5p/ITGA6 signal axis in Hirschsprung’s disease

**DOI:** 10.48101/ujms.v126.7895

**Published:** 2021-08-20

**Authors:** Chuancheng Sun, Bing Xu, Liang Wang, Yilin Su

**Affiliations:** Pediatric Surgery, The First Affiliated Hospital of China University of Science and Technology (Anhui Provincial Hospital), Hefei, Anhui, China

**Keywords:** LncRNA DRAIC, miR-34a-5p, ITGA6, Hirschsprung’s disease, proliferation, migration

## Abstract

**Background:**

Hirschsprung’s disease (HSCR) is a common defect in newborns, and studies have revealed that long non-coding RNA (lncRNA) is involved in the progression of HSCR. This research study aims to investigate the mechanism of downregulated RNA in cancer (DRAIC) on cell proliferation and migration in HSCR.

**Methods:**

Quantitative reverse transcription–polymerase chain reaction (qRT-PCR) was used to detect the expression of DRAIC in HSCR bowel stenosis tissues and normal colon tissues. Cell-counting kit-8 (CCK-8) and Transwell assays were employed to explore whether cellular functions change after overexpression or knockdown of the DRAIC in SH-SY5Y cells and human 293T cells. Protein expression levels were determined by Western blot analysis. RNA pull-down and dual-luciferase reporter assays were used to confirm the competitive relationship of DRAIC and integrin subunit alpha 6 (ITGA6) through their association with miR-34a-5p.

**Results:**

The lncRNA DRAIC was significantly increased in colon tissue from HSCR patients. The overexpression of DRAIC inhibited SH-SY5Y cell and human 293T cell proliferation and migration. Knockdown of DRAIC, however, promoted cell proliferation and migration. The RNA pull-down and dual-luciferase reporter assays have proven the competitive relationship between DRAIC and ITGA6 through their association with miR-34a-5p. Further rescue experiments have confirmed that DRAIC regulates cell proliferation and migration by affecting the miR-34a-5p/ITGA6 signal axis in HSCR.

**Conclusion:**

DRAIC promoted cell proliferation and migration by regulating the miR-34a-5p/ITGA6 signal axis in HSCR.

## Introduction

Hirschsprung’s disease (HSCR) is one of the most common congenital intestinal diseases reported in children. Its pathogenesis includes loss or dysfunction of enteric neural crest cells (ENCCs) in the distal bowel, leading to the lack of parasympathetic ganglion cells in the submucosa and myenteric plexus of the intestinal tract (1). The incidence rate of this disease is about 1/5,000 in newborns, with the male: female ratio being 4:1 (2); however, until now, the molecular pathological mechanism of HSCR remains unclear.

Long non-coding RNAs (lncRNAs) are a kind of functional RNAs with lengths exceeding 200nt in eukaryotes. It regulates gene expression at the epigenetic, gene transcription and post-transcriptional level, and then affects the evolution of disease. Previous studies have shown that lncRNA play a key role in the regulation of the incidence and development of HSCR (3, 4). Downregulated RNA in cancer (DRAIC, Gene ID: 145837) is a lncRNA whose coding gene is located on the chromatin of 15q23, which is abnormally expressed in a variety of malignant tumors (5–7). Niu et al. (8) found that DRAIC is highly expressed in HSCR patients; however, their effect on the pathogenesis of HSCR should be explored further.

Integrin subunit alpha 6 (ITGA6), a coding integrin α6 subunit, is a specific receptor of laminin. Previous studies have shown that integrin and laminin exist in the intestinal microenvironment, and play an important role in the migration of ENCCs and the progression of HSCR (9,10). ITGA6 has been reported to be upregulated in HSCR patients; however, the function of ITGA6 in HSCR and its potential molecular mechanism are unclear. Meanwhile, by a HSCR pathway-related co-expressed network, Niu et al. (8) proved that DRAIC and ITGA6 were co-expressed, and ITGA6 was involved in the HSCR-related Kyoto Encyclopedia of Genes and Genomes (KEGG) pathway. Recently, some lncRNAs were used as competing endogenous RNA (ceRNA) in the incidence of HSCR (11–13). Thus, we hypothesized that DRAIC, used as a ceRNA, regulates the expression of ITGA6 and affects the development of the enteric nervous system.

Previous studies have revealed that miR-34a-5p participated in the regulation of Alzheimer’s disease (14), cerebral ischemia, and reperfusion (15), and was involved in the differentiation of neural crest cells (16). In addition, low expression of miR-34a-5p results in the development of colorectal cancer by promoting the migration of colorectal cancer cells (17), with LncARSR promoting invasion and metastasis of colorectal cancer by sponging miR-34a-5p (18). These data suggested that miR-34a-5p may be associated with the nervous system, as well as the digestive system.

This study aimed at identifying the effects and mechanisms of DRAIC on cell proliferation and migration in HSCR to provide a new direction for the development of HSCR treatment.

## Materials and methods

### Clinical samples

Tissues samples were obtained from 18 children with HSCR, and 18 matched controls were obtained from children with surgical treatment. The collected tissues were diagnosed after surgery. All tissue samples were stored at −80°C after intestinal resection. The study was approved by the Ethics Committee of The First Affiliated Hospital of University of Science and Technology of China (Anhui Provincial Hospital). Parents signed an informed consent form for their children’s participation.

### Cell culture and transfection

For *in vitro* experiments, human 293T and SH-SY5Y cell lines were cultured in Dulbecco’s Modified Eagle Medium (HyClone) supplemented with 10% fetal bovine serum (FBS), 100 U/mL penicillin, and 100 µg/mL streptomycin at 37°C with 5% CO_2_. The small interfering RNA against DRAIC (si-DRAIC, specific RNA interfere sequences for target gene (siRNA) as control), miR-34a-5p mimics (mimic NC as control), and overexpression plasmid of DRAIC and ITGA6 (vector as control) were obtained from GenePharma (Shanghai, China). The 293T and SH-SY5Y cells, which were cultured to 50–60% confluence, were transfected with the overexpression plasmid or siRNA using Lipofectamine 2000 (Invitrogen).

### Quantitative reverse transcription – polymerase chain reaction

The total RNA of each sample and cell lines were extracted using TRIzol reagent (Life Technologies, Carlsbad, CA), and the RNA was reverse transcribed into cDNA using a random primer method. The premix, cDNA templates, and upstream and downstream primers of LncRNA DRAIC were added according to TaqMan reagent instructions (Applied Biosystems, Foster City, CA). The LncRNA DRAIC: F:5’-TCCCACGATGATCCTGAGGT-3’, R:5’-TGTTCCACAACGTCCTCACC-3’. The GAPDH: F:5’-GAAGATGGTGATGGGATTTC-3’, R:5’-GAAGGTGAAGGTCGGAGTC-3’. The relative expression levels of the target genes were examined using the 2^-ΔΔCt^ method.

### Cell counting kit-8

Cells (1 × 10^4^ cells/mL) were cultured in 96-well plates, and after 24 h, 10 µL of CCK8 (Beyotime) were added to each well. Then we used the TECAN infinite M200 Multimode microplate reader (Tecan, Mechelen, Belgium) to measure the optical density (OD) of the cells at 450 nm. All assays were performed three times independently.

### Transwell

The cells were seeded in the upper chamber of the Transwell membrane and cultured for 48 h at 37°C with 5% CO_2_. Then the cells were fixed in methanol, stained with a crystal violet solution, and washed in phosphate buffer saline. The cells were counted from five fields randomly under the microscope. The experiment was repeated three times.

### RNA pull-down assay

A DNA fragment containing the full-length DRAIC sequence or a negative control sequence was polymerase chain reaction (PCR) amplified using T7 RNA polymerase (Roche, Basel, Switzerland). The plasmid DNA was linearized using the restriction enzyme XhoI. Biotin-labeled RNA was reverse transcribed using Biotin RNA Labeling Mix (Roche) and T7 RNA polymerase (Takara Biomedical Technology). The products were treated with RNase-free DNase I (Roche) and purified using an RNeasy mini kit (Qiagen, MD, USA), with the resulting RNA used for real-time PCR assays.

### Dual-luciferase reporter assay

The targets of DRAIC were predicted by Starbase 2.0. The binding sites between miR-34a-5p and ITGA6 were predicted by the TargetScan database. The DRAIC wild-type (WT), DRAIC mutant (Mut), ITGA6 3’UTR WT, or ITGA6 3’UTR Mut reporters were constructed using the psiCHECK-2 vectors (Promega). The 293T cells were co-transfected with a reporter and miR-34a-5p mimic or mimic negative control (NC) with the Lipofectamine2000 reagent. After 48 h, the luciferase activities were monitored using the luciferase reporter assay kit (Promega).

### Western blot analysis

Total protein was extracted from the cells by Radio Immunoprecipitation Assay (RIPA) lysis buffer and a protease inhibitor mix. Protein concentrations were measured using a bicinchoninic acid (BCA) protein assay kit. Equal amounts of total protein were separated with SDS-PAGE (sodium dodecyl sulfate-polyacrylamide gel electrophoresis), and then the separated proteins were transferred to polyvinylidene fluoride membranes. The membranes were blocked with 5% no-fat milk for 2 h at room temperature. The membranes were incubated with ITGA6 antibody (Ab235905, Abcam). α-Tubulin was used as control. After a triple wash with tris-buffered saline with Tween 20 (TBST) for 10 min, the membranes were incubated with Horseradish Peroxidase (HRP)-conjugated secondary antibodies at room temperature for 2 h. Then, the membranes were developed using the electrochemiluminescence (ECL) system (Millipore, MA). The intensity of the bands was quantified using Gel‑Pro Image Analysis software.

### Statistical analysis

Data are expressed as mean ± standard deviation (SD) for each group. SPSS 22.0 software (Chicago, IL, USA) was used for statistical analysis. An independent sample t-test was used for comparison between the two groups. One-way analysis of variance was used to analyze the obtained data among multiple groups. *P* < 0.05 was considered to be statistically significant.

## Results

### The DRAIC was upregulated in HSCR

**Figure 1:**
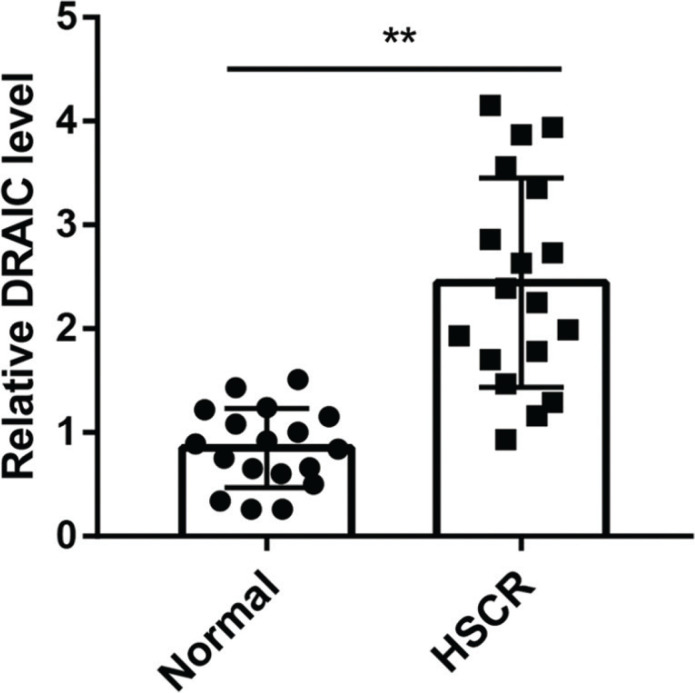
DRAIC is up-regulated in HSCR bowel stenosis tissues. The expression of DRAIC in HSCR bowel stenosis tissues (*n* = 18) and normal colon tissue (*n* = 18). DRAIC was significantly increased in HSCR compared with normal. Data are reported as mean ± SD. **P < 0.01 versus normal group.

To study the relationship between DRAIC and HSCR, we compared the expression of DRAIC in HSCR bowel stenosis tissues and normal colon tissue. The expression of DRAIC in HSCR bowel stenosis tissues was significantly higher than that in normal tissues (Figure 1).

### Overexpression of DRAIC inhibits cell proliferation and migration in HSCR

**Figure 2:**
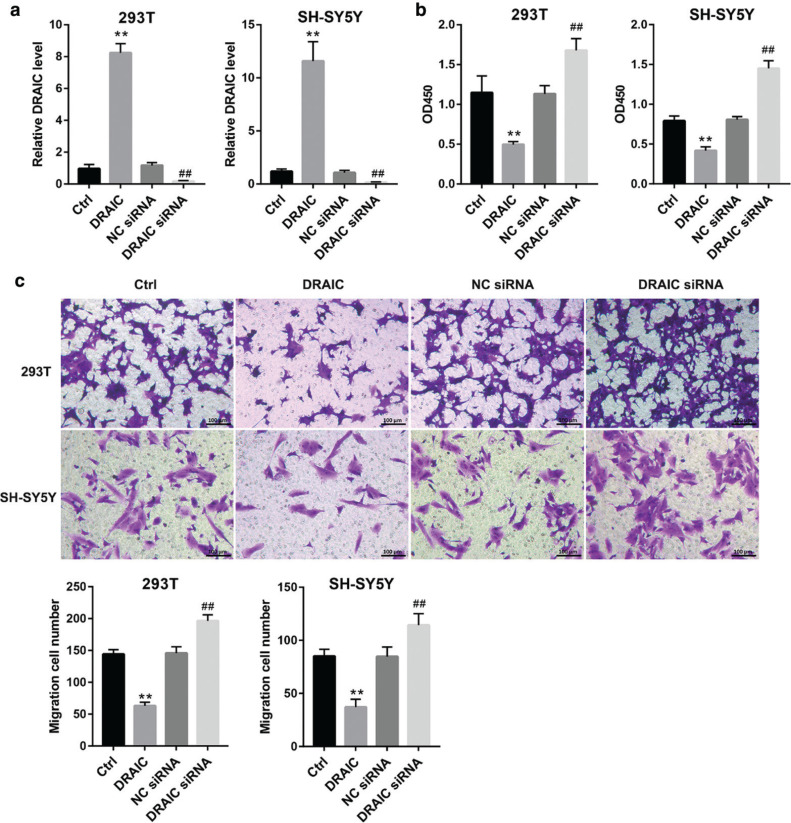
Overexpression of DRAIC inhibits cell proliferation and migration in HSCR The293T and SH-SY5Y cells were transfected with DRAIC overexpression plasmid or targeted interference sequence, respectively. (a) The overexpression and knockdown efficiency of DRAIC were detected by qRT-PCR. (b) The cell proliferation was measured by CCK-8 kit. (c) The cell migration was analysed by Transwell assay. Data are expressed as mean ± SD. **P < 0.01 versus Ctrl group. ##*P* < 0.01 versus NC siRNA group.

To study the effect of DRAIC on cell proliferation and migration in HSCR, the DRAIC overexpressing plasmid and interference sequence were transfected into 293T and SH-SY5Y cells. The DRAIC overexpressing plasmid significantly increased the expression of DRAIC, and DRAIC interference sequence obviously reduced the expression of DRAIC (Figure 2a). The results of the CCK8 assay revealed that overexpression of DRAIC inhibited cell proliferation, and DRAIC silencing promoted cell proliferation (Figure 2b). Similarly, the Transwell assay data proved that overexpression of DRAIC inhibited cell migration, while DRAIC silencing promoted cell migration (Figure 2c).

### DRAIC regulated the expression of ITGA6 in cells by sponging miR-34a-5p

**Figure 3:**
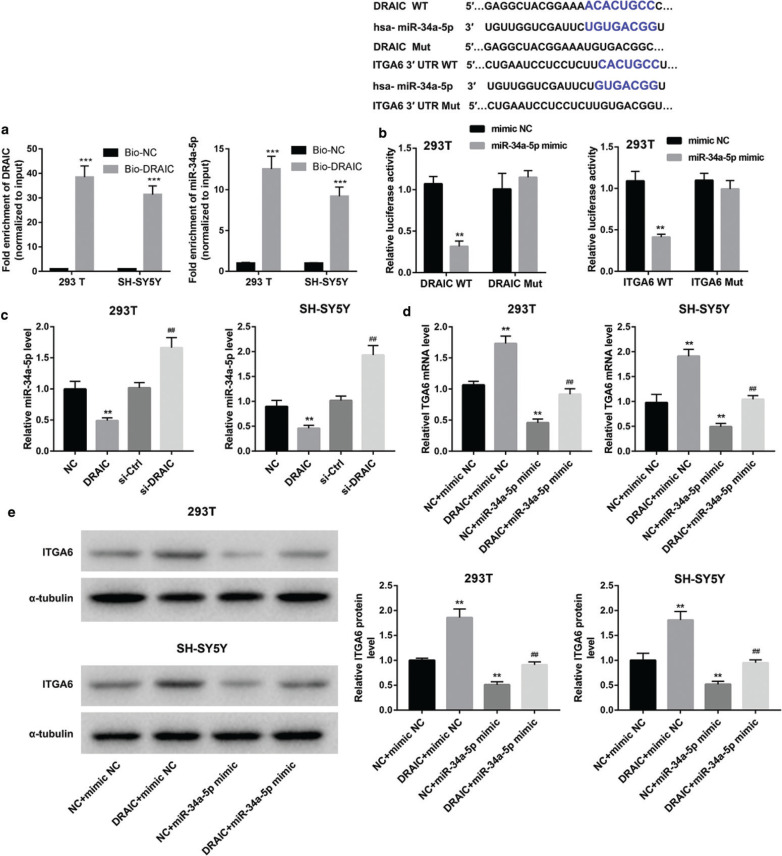
DRAIC regulated the expression of ITGA6 in cells by sponging miR-34a-5p. (a) RNA pull-down assay was used to identify the relationship between the miR-34a-5p and DRAIC. (b) Luciferase activity experiment proved that miR-34a-5p and DRAIC had binding sites, and miR-34a-5p directly bind to ITGA6 3’UTR. (c) The expression level of miR-34a-5p was detected by qRT-PCR in 293T and SH-SY5Y cells transfected with DRAIC overexpression plasmid or interference sequence. (d, e) mRNA and protein expression levels of ITGA6 were determined by qRT-PCR and Western blot, respectively. The blue text shows the binding site of the two genes. Data are reported as mean ± SD. **P < 0.01, ***P < 0.001 versus Bio-NC or mimic NC and/or NC group. ##*P* < 0.01 versus DRAIC + mimic NC group.

Through online website Starbase V2.0, miR-34a-5p and DRAIC had binding sites. RNA pull-down and dual luciferase reporter gene assays were used to verify the relationship between the miR-34a-5p and DRAIC. The results of biotin-labeled pull-down assay revealed a significant amount of DRAIC and miR-34a-5p in the DRAIC pull-down pellet compared with that observed in the control group as measured by qPCR (Figure 3a). These data suggested that DRAIC could negatively regulate miR-34a-5p expression in 293T and SH-SY5Y cells. In addition, the luciferase reporter gene results demonstrated that miR-34a-5p mimic remarkably reduced the luciferase activity of pmirGLO-DRAIC-wt but not pmirGLO-DRAIC-mut (Figure 3b).

Next, the results of quantitative reverse transcription--PCR revealed that the expression of miR-34a-5p was significantly decreased in the DRAIC overexpression group, while increased in the DRAIC silencing group, which further proved that DRAIC could negatively regulate the expression of miR-34a-5p in 293T and SH-SY5Y cells.

Through online website TargetScan, miR-34a-5p and ITGA6 had binding sites. We also performed the dual-luciferase reporter gene to verify the relationship between the miR-34a-5p and ITGA6. The results demonstrated that miR-34a-5p mimic remarkably reduced the luciferase activity of pmirGLO-ITGA6-wt but not pmirGLO-ITGA6-mut (Figure 3c). These data suggested that miR-34a-5p could negatively regulate ITGA6 expression in 293T and SH-SY5Y cells. To further prove that DRAIC affects the expression of ITGA6 through miR-34a-5p, 293T and SH-SY5Y cells were co-transfected with DRAIC overexpression plasmid and miR-34a-5p mimic. The mRNA and protein expression of ITGA6 were increased by DRAIC overexpression, which was reversed by miR-34a-5p mimic (Figure 3d and e). These results revealed that DRAIC regulated the expression of ITGA6 by sponging miR-34a-5p.

### DRAIC inhibits cell proliferation and migration by regulating the miR-34a-5p/ITGA6 signal axis

**Figure 4:**
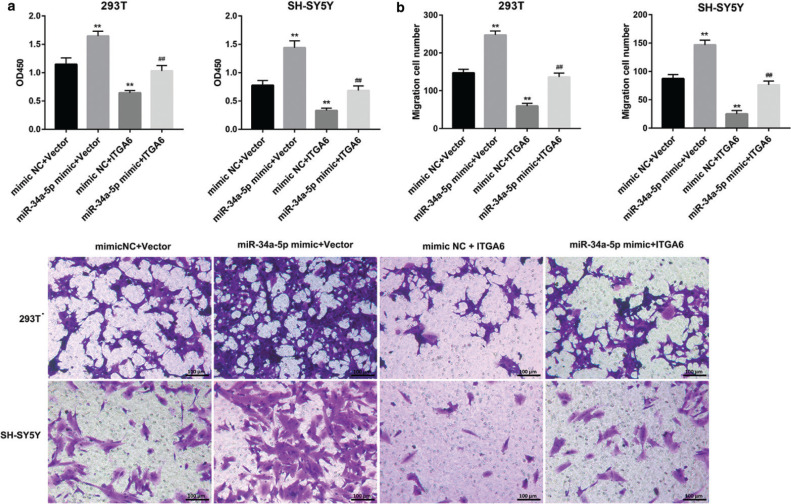
DRAIC inhibits cell proliferation and migration by regulating the miR-34a-5p/ITGA6 signal axis. 293T and SH-SY5Y cells were co-transfected with miR-34a-5p mimic and ITGA6 overexpression plasmid. (a) Cell proliferation was measured by CCK-8 assay. (b) Cell migration was analyzed by Transwell assay. Data are reported as mean ± SD. **P < 0.01, versus mimic NC +vector group. ##*P* < 0.01 versus miR-34a-5p mimic +vector group.

To study whether DRAIC inhibits cell proliferation and migration by regulating the miR-34a-5p/ITGA6 signal axis, 293T and SH-SY5Y cells were co-transfected with miR-34a-5p mimic and ITGA6 overexpressing plasmid. The results of CCK8 assay revealed that miR-34a-5p mimic promoted cell proliferation, while the ITGA6 overexpressing plasmid inhibited cell proliferation. Meanwhile, overexpression of ITGA6 reversed the effect of miR-34a-5p mimic on cell proliferation (Figure 4a). In accordance with the trend of cell proliferation, the miR-34a-5p mimic promoted cell migration, while the ITGA6 overexpressing plasmid inhibited cell migration. Meanwhile, overexpression of ITGA6 reversed the effect of miR-34a-5p mimic on cell migration (Figure 4b). The study results revealed that DRAIC inhibits cell proliferation and migration by regulating the miR-34a-5p/ITGA6 signal axis in HSCR.

## Discussion

HSCR is a congenital malformation of gastrointestinal tract reported in newborns. Enteric neurons play an important role in advancing intestinal motility. Because of the lack of enteric neurons, the patients suffer from abdominal enlargement and constipation, which even threaten their lives. Until now, several lncRNAs have been reported to be abnormally expressed in colon tissues of HSCR patients, which play an important regulatory role in the progression of HSCR (19, 20). In this study, DRAIC was found to be highly expressed in colon tissues of HSCR patients, which was consistent with the results reported in a previous article (8).

We found that overexpression of DRAIC inhibited cell proliferation and migration, while knockdown of DRAIC had the opposite effect. These results confirmed that DRAIC could inhibit cell proliferation and migration in HSCR. Liao et al. (21) reported that DRAIC was significantly increased in nasopharyngeal carcinoma tissues. Levels of DRAIC expression were related to the clinical stages of nasopharyngeal carcinoma patients. Functional assays revealed that DRAIC acts as a miR-122 sponge to facilitate nasopharyngeal carcinoma cell proliferation, migration, and invasion via regulating SATB homeobox 1 (SATB1). Li et al. (5) have demonstrated that DRAIC was highly expressed in esophageal cancer cells and acts as a miR-149-5p sponge to facilitate esophageal cancer cell autophagy by regulating nuclear factor I B (NFIB). Research studies indicated that the miR-34 family was down-regulated in most of the cancer cells. MiR-34a-5p, which derives from miR-34a, is expressed at a low level in a variety of cancers, whereas overexpression of miR-34a-5p increased apoptosis (22–24). As miR-34a-5p and DRAIC had binding sites predicted by Starbase V2.0, we performed luciferase activity test and RNA pull-down assay to prove that miR-34a-5p directly binds to DRAIC, and DRAIC negatively regulate the expression of miR-34a-5p.

It has been reported that ITGA6 is the target gene of several miRNAs (25, 26), and several lncRNAs could act as a ceRNA of miRNA to regulate the expression of ITGA6 (27, 28). As DRAIC and ITGA6 were co-expressed, and ITGA6 was involved in the HSCR-related KEGG pathway, we hypothesized that DRAIC, as a ceRNA, regulated the expression of ITGA6 in HSCR. In this study, using a dual-luciferase reporter assay, we were able to prove that ITGA6 was a target of miR-34a-5p. Besides that, co-transfection experiments showed that DRAIC regulated the expression of ITGA6 by sponging miR-34a-5p. Finally, rescue experiments further proved that DRAIC influenced cell proliferation and migration by regulating the miR-34a-5p/ITGA6 signal axis.

In summary, our research study confirmed a novel mechanism in the pathogenesis of HSCR. The lncRNA DRAIC could regulate cell migration and proliferation by regulating the miR-34a-5p/ITGA6 axis in congenital megacolon disease. However, to explore the potential mechanisms of HSCR, the best cell model is still the ENCCs, and we are currently incapable of propagating such an ideal cell line for our studies. More studies are needed to further determine the functions of the lncRNAs in the incidence of HSCR.
